# Experimentally measured group direct benefits according to worker density explain group living of the termite *Reticulitermes chinensis*

**DOI:** 10.1002/ece3.7685

**Published:** 2021-06-16

**Authors:** Zhuangdong Bai, Yibin Liu, David Sillam‐Dussès, Rui‐Wu Wang

**Affiliations:** ^1^ School of Ecology and Environment Northwestern Polytechnical University Xi’an China; ^2^ Laboratory of Experimental and Comparative Ethology UR4443 University Sorbonne Paris Nord Villetaneuse France

**Keywords:** biomass accumulation, group living, reproduction, *Reticulitermes chinensis*, worker density

## Abstract

The evolution of cooperation requires more benefits of group living than solitary lifestyle. However, to some degree, our understanding about the benefits is hindered by abstract debates over theoretical and experimental evidences of individual selection or group selection because it is difficult to examine the actual benefits at the group level. Moreover, group density is a crucial ecological factor which deeply affects group reproduction and survival, few studies have been performed in social insects. Here, we study the effects of worker density on group direct benefits in the termite species *Reticulitermes chinensis*. The termite *R*. *chinensis* is an ideal model which lives with a high worker density in wood. We used the quantity of eggs and the total biomass (biomass of all group members) accumulation as two components of group benefits. We investigated the group benefits in the context of worker density according to eleven worker densities, and we measured the group benefits and the resource consumption with the same group members in two types of artificial nest areas. Moreover, we counted the stomodeal trophallaxis occurrences from any workers to queens under three worker densities to explore the degree of cooperation according to worker density. We found that both the number of eggs and the total biomass accumulation significantly increased with increasing worker density in groups. Furthermore, the consumption of resources was similar between groups with the same number of individuals gathered in small or large nest areas, but the production of eggs and the biomass accumulation were higher in groups of small nest areas than in large nest areas. Additionally, we found the stomodeal trophallaxis behavior significantly increased in higher worker density groups. Our results suggest that the group benefits influenced by the high worker density may at least partially explain the group living of eusocial insects in ecology.

## Graphical abstract

Worker density is a crucial ecological factor which deeply affects group reproduction and group biomass. Therefore, group direct benefits supported by worker density could explain the group living of termites and probably of other eusocial insects.

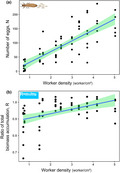

## 1 INTRODUCTION

Shifting from solitary lifestyle to group living is considered to be one of the major evolutionary transitions during evolution process (Boomsma & Gawne, 2018; Szathmáry & Smith, 1995; West et al., 2015; Wilson & Wilson, 2007). According to literature, group living can be found in many animal taxa in various ways, such as individuals nesting and feeding near each other assembled by simple mutual attraction between them (Krause et al., 2002), or individuals which may temporarily gather parents and offspring at the same time, or individuals in permanent societies with reproductive division of labor in eusocial societies (Wilson, 1971). However, whether to measure direct benefits at the individual level or at the group level is a matter of great debate (Smallegange & Egas, 2015).

Conventionally, it is considered that the ecological success of group living species relies on the benefits provided to individuals, such as improved defense against predators, enhanced foraging efficiency and higher survival rate, or/and increased reproductive success (Bilde et al., 2007; Janson & Goldsmith, 1995; Majolo et al., 2008; Morand‐Ferron & Quinn, 2011). However, during evolutionary transitions in individuality, some groups of individuals become so integrated that they evolve into a new higher level, and some individuals among the group specialized in reproductives while other individuals specialized in somatic functions (Szathmáry & Smith, 1995; West et al., 2015). The initial fitness benefits are considered at the individual level, of which include the components of individual survival and individual reproduction (Avilés & Tufino, 1998), but they are controversial to explain these evolution occurrences (Michod & Nedelcu, 2003; Queller, 1997).

In the context of the colony formation, such as the appearance of eusocial insects, the individuals cooperate in complex ways toward the common goal of the success of the colony. Wheeler proposed that such colonies can be regarded as superorganisms when they have morphologically differentiated reproductive and nursing castes (Wheeler, 1911). In recent years, multilevel selection theory (including group selection) have been revived (Wilson & Wilson, 2007; Wilson & Hölldobler, 2005), and most evolutionary biologists agree that selection at the group level can produce cooperative traits with group‐level benefits (Gardner, 2015; Lehmann et al., 2007; Lion et al., 2011; Marshall, 2011). However, with some individuals giving up their own lifetime reproductive potential to raise the offspring of others, the group‐level benefits become gradually “decoupled” from that of its constituent individuals. Although the MVSHN index (named by the authors: Michod, Viossat, Solari, Hurand, and Nedelcu) captures the benefits to the group from the reproductive‐somatic division of labor (Michod et al., 2006), few experimental studies have been designed to measure group direct benefits. Moreover, in eusocial insects, how variable density influences group benefits is unclear.

The density and the size are the key aspects of the colony that have pronounced effects on the information sharing and the cooperation activities, so that to have important benefit consequences for colony members (Avilés & Tufino, 1998; Billick, 2001; Rubenstein & Wrangham, 1986). Some previous studies showed that large group size may be beneficial for survival (for example, in terms of predation avoidance, or a buffered environment within a group; Treherne & Foster, 1982), as well as for reproduction (e.g., in terms of a higher number or better quality of offspring; Strohm & Bordon‐Hauser, 2003), but the effect of density is not well addressed.

Besides, the other important question is how specialization of individuals combined with worker caste density takes effect in group living of social insects. The basic form of specialization involves the separation of reproductives and worker castes in eusocial insects. The specialization of the group members may help to enhance the benefits with higher worker density (Wilson, 1975). Hence, we used a series of groups of the termite species *Reticulitermes chinensis* to examine the effects of worker density on the direct benefits of the colony. Colonies of *R*. *chinensis* live naturally with high worker density and large groups in dead wood. One primary queen and king and/or a few supplementary reproductives perform reproduction, while workers and soldiers act as helpers to defend the nest from predators and parasitoids or to ensure foraging food, building nest, and rearing brood (Li et al., 2010). Here, we used the total biomass accumulation and the quantity of eggs laid as the measurement of the group direct benefits. We experimentally measured these two variables with an increased worker density to explore the influence of worker density on the group direct benefits.

## 2 MATERIALS AND METHODS

### 2.1 Collection and maintenance of groups

Ten *Reticulitermes chinensis* colonies were collected from dead pine trees in the Botanical Garden at Chengdu, Sichuan Province, China in 2018. We selected seven colonies with the large number of termites as our experimental objects. Each colony was divided into 11 groups, and each group contained 300 individuals. These 300 individuals were distributed into 15‐cm Petri dishes. The other three colonies with the small number of termites were divided into 23 groups for substitute objects. The bottom of each Petri dish was covered with one moist piece of filter paper for providing food and water. All groups were kept in the climatic chamber (25°C, 70% relative humidity, 12 hr day/night cycle). One new piece of filter paper and 1 ml water were added in each Petri dish every week.

After 1 month, one or more supplementary queens were differentiated in each group, and all but one supplementary queen and one supplementary king were removed from each group. Food and water were supplied to the groups in the same way. These groups were used for the following experiments.

### 2.2 Worker density‐dependent experiments

We estimated the influence of worker density on group benefits with two artificial sets: set A with 25, 50, 75, 100, 125, and 150 group members in 6‐cm Petri dish (with five repetitions of each treatment corresponding to five colonies, 30 groups in total); set B with 50, 100, 150, 200, and 250 group members in 9‐cm Petri dish (with seven repetitions of each treatment corresponding to seven colonies, 35 groups in total). The values of worker densities were 0.72, 0.78, 1.48, 1.63, 2.23, 2.48, 2.99, 3.32, 3.74, 4.17, and 5.02 worker/cm^2^ (Table S1). Each Petri dish was covered at the bottom with a piece of filter paper, as the only available food source for termites. Before introduction of the termites into the Petri dishes, we weighed the dry pieces of filter paper, the biomass of each queen, and the total biomass of each group (workers, soldiers, king, and queen). Then, each of these groups was kept for 25 days at 25°C, 70% relative humidity and with a 12 hr day/night cycle. At the beginning of the experiment, each group received 250 µl of water. Then, for the first 10 days, 200 µl of water were added every 5 days; for the next 15 days, 200 µl of water were added every 7.5 days. At the end of the experiment, the remaining pieces of filter paper (from which we removed feces) were dried in an oven at 37°C for 60 min. We weighted the dry remaining pieces of filter paper, as well as the queen and the total termite biomass of each group. At the same time, the number of eggs in each group was counted.

### 2.3 Cooperation behavior experiments

In order to investigate how the worker density influences the level of cooperation behavior within groups, we studied the number of occurrences of stomodeal trophallaxis performed by any workers to the queen in each group. In this purpose, we made three treatments (23 workers, one soldier, one queen, one king in 9‐cm Petri dish, in 6‐cm Petri dish, or in 3.5‐cm Petri dish; the worker densities were 0.36, 0.81, and 2.39 worker/cm^2^, respectively), and each treatment had 12 replicates from three colonies (each colony had four replicates). Then, we kept all these treatment groups in the same conditions of temperature, humidity, and light for 5 days to stabilize the groups in their new environment. Last, the stomodeal trophallaxis occurrences were recorded and counted from each group for 2 hr by using a camera SONY AX100E.

### 2.4 Statistical analysis

Firstly, we defined the ratio of total biomass accumulation of each group, *R*, as $R=\frac{m_{1}}{m_{2}}$. *m*_1_ and *m*_2_ were the fresh weight of the total group members at the end and at the beginning of the experiment, respectively. The number of eggs, *N*, was used to measure the group reproduction. The reduction of the dry weight of the filter papers was used to measure the food consumption of the group, with the equation: Δ*W* = *W*_1_ − *W*_2_. *W*_1_ and *W*_2_ refer to the dry weight of the filter paper at the beginning and at the end of the experiment, respectively. We defined *T* as the number occurrences of stomodeal trophallaxis behavior within 2 hr.

Duo to the random effects of different colonies in each treatment, multivariate analyses were performed using linear mixed models (LMMs; Bolker et al., 2009) to determine statistical significance for the influence of four main effects (worker density, queen biomass at the beginning, group number, queen biomass at the end) on the number of eggs. Because the body size of the queen may have an effect on the fecundity, the biomass of the queen at the beginning and at the end of the experiments were considered as two effects. In the same way, LMMs were applied for the influence of two main effects (worker density and group number) on the ratio of total biomass accumulation. Data of the food consumption and group benefits between the same group members in the two types of artificial nests were compared by using one‐way ANOVA. Data of the stomodeal trophallaxis among the three types of worker densities were analyzed by one‐way ANOVA followed by Tukey's HSD test. All statistical analyses were performed in the IBM SPSS Statistics 20 and the program R v3.6.3 (R Core Team, 2020, see http://www.R‐project.org/). In R, the packages of *lme4*, *lattice and ggplot2* were used.

## 3 RESULTS

### 3.1 Influence of worker density on group benefits

**FIGURE 1:**
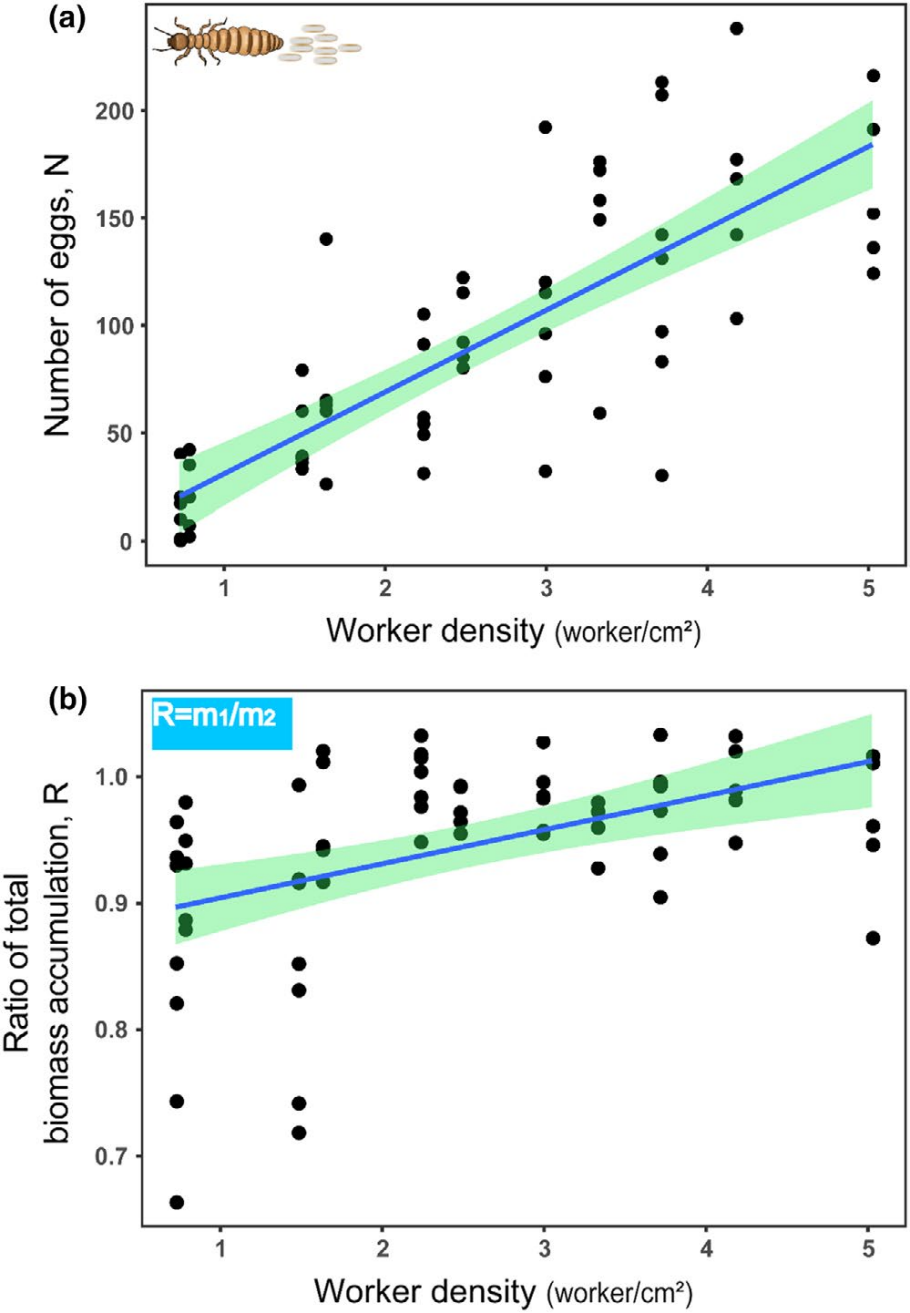
Number of eggs (a) and ratio of total biomass accumulation (b) according to worker density in our experiments in the termite *R. chinensis*. The ratio of total biomass accumulation, R, was calculated with the total biomass per group at the end (*m*_1_) and at the beginning of the experiment (*m*_2_). Both the number of eggs and the ratio of total biomass accumulation increased with increasing worker density. Solid lines denote predicted relationships of least‐squared means from LMMs, and green shaded areas represent 95% confidence intervals expected from LMMs

We firstly analyzed the influence of four effects to the group fertility. We found that the number of eggs was not significantly affected by the group number (LMMs: *t* = −0.104, *p* = .918, *n* = 65), by the biomass of the queen at the beginning of the experiments (LMMs: *t* = −0.656, *p* = .514, *n* = 65) or by the biomass of the queen at the end of the experiments (LMMs: *t* = 1.797, *p* = .077, *n* = 65), but only significantly affected by the worker density (LMMs: *t* = 4.667, *p* = .012, *n* = 65; Table S2). As the worker density increasing, the number of eggs increased linearly (Figure 1a). Then, we analyzed the influence of worker density and group number to the ratio of total biomass accumulation. We found that the ratio of total biomass accumulation was not significantly affected by the group number (LMMs: *t* = 0.622, *p* = .537, *n* = 65), but by the worker density (LMMs: *t* = 2.596, *p* = .012, *n* = 65; Table S3). Also, the ratio of total biomass accumulation increased linearly with the increasing worker density (Figure 1b). Our experimental results demonstrate that increased worker density of *R*. *chinensis* does not only improve the fertility of the group, but also enhances the overall biomass accumulation of the group.

### 3.2 Comparison of group consumption and group benefits

**FIGURE 2:**
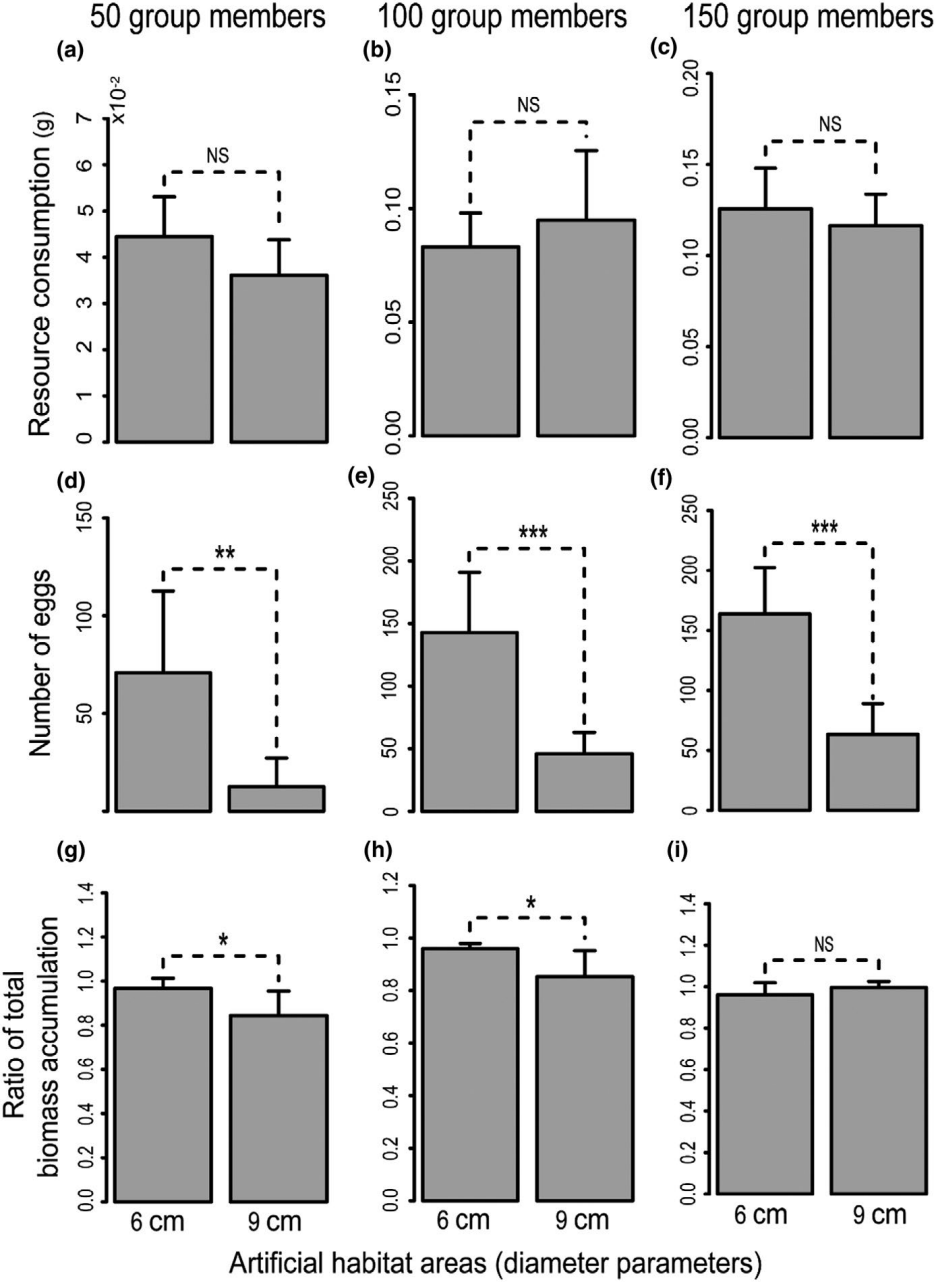
Comparison of the resource consumption (a, b, and c), the eggs production (d, e, and f), and the ratio of total biomass accumulation (g, h, and i) in two artificial nests of *R. chinensis* during 25 days. The artificial nests are two types of Petri dishes (6‐cm or 9‐cm diameter) containing three kinds of group individuals (50, 100, or 150). Overall, there is no significant difference in the resource consumption between groups in 6‐ or 9‐cm Petri dishes (whatever the number of individuals is) but groups in smaller dishes produced more eggs than groups in larger dishes (whatever the number of individuals is) and groups in smaller dishes accumulated more biomass than groups in larger dishes with 50 and 100 individuals in the group (but no difference was found in 150 individuals in the group). NS, not significant; *p ≤ .05; **p ≤ .01; ***p ≤ .001

Our above‐mentioned results demonstrate that the termite *R*. *chinensis* remarkably increased group benefits along with the worker density. However, we wonder whether the increased group benefits in experiments with high worker density result from the fact that workers consumed more resources or that workers efficiently improved the utilization of resources. Hence, we directly measured the resource consumption, the number of eggs, and the total biomass accumulation in two types of artificial nests with three different group members. The results showed that group members with 50 individuals consumed the same quantity of filter papers in 6‐cm Petri dish than in 9‐cm Petri dish (*F* = 3.158, *p* = .106, Figure 2a), but had a higher ratio of the total biomass accumulation (*F* = 5.35, *p* = .043, Figure 2g), and had a higher number of eggs (*F* = 11.93, *p* = .006, Figure 2d) in 6‐cm Petri dish than in 9‐cm Petri dish. The same results were obtained for groups made of 100 group members, that is, the consumption of filter paper was the same, but the number of eggs and the ratio of total biomass accumulation were higher in groups of 100 group members in 6‐cm Petri dish than in 9‐cm Petri dish (*F* = 0.630, *p* = .446, Figure 2b; *F* = 24.89, *p* = .001, Figure 2e; *F* = 5.49, *p* = .041, Figure 2h, respectively). For 150‐member groups, the difference of consumption of filter paper and the ratio of total biomass accumulation were not significantly different between them in 6‐cm Petri dish and in 9‐cm Petri dish (*F* = 0.663, *p* = .434, Figure 2c; and *F* = 1.98, *p* = .19, Figure 2i, respectively), but the number of eggs was higher in 6‐cm Petri dish than in 9‐cm Petri dish (*F* = 29.74, *p* = .0001, Figure 2f). Overall, the termite *R*. *chinensis* did not consume resources differently when the same number of individuals in the different type's Petri dish, but it produced more group benefits in the areas with the higher worker density.

### 3.3 Degree of cooperation under different worker densities

**FIGURE 3:**
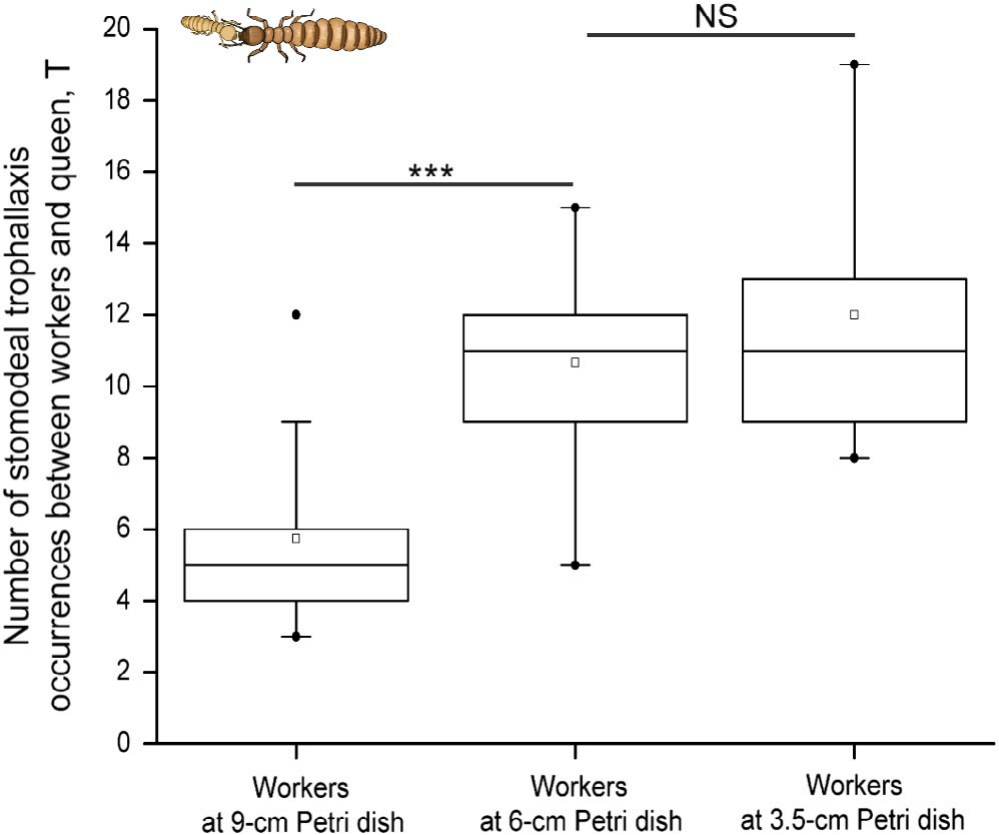
Number of stomodeal trophallaxis occurrences between any 23 workers and the queen in artificial groups of *R. chinensis* with three different worker densities. Workers showed more stomodeal trophallaxis behavior to the queen when the worker density was medium or high in comparison with low density, but no significant difference between the medium and the high densities. Box plots are shown with quartiles, average, minimum, and maximum values. NS, not significant; ***p ≤ .001

A previous study has suggested that the trophallaxis behavior is one of the typical cooperation behaviors among different castes in termites (Suárez & Thorne, 2000). Lower termites have a strict division of labor, queens are specialized in reproduction while workers primarily maintain the colonies. The nutritional materials obtained by queens mainly come from the workers by using stomodeal trophallaxis and proctodeal trophallaxis behaviors (LaFage & Nutting, 1978). Our above results showed that more eggs were laid by queens with the increasing worker density. It is not hard to guess that the nutritional material supplies were more needed for queens in order to lay more eggs. Thus, we hypothesize that the number of trophallaxis occurrences from workers to queens could reflect the level of cooperation between workers and reproductive castes, and it could be influenced by different worker densities. We recorded the number of stomodeal trophallaxis occurrence at three level of worker densities and found it significantly higher at medium density than at low density (Tukey's HSD test: *p* = .001, Figure 3), but it did not show significant difference between medium and high densities (Tukey's HSD test: *p* = .532, Figure 3).

## 4 DISCUSSION

Most social species, which live in groups (Bourke, 2011; Krause et al., 2002; Wilson, 1971), have dominated the terrestrial ecosystems by their biomass and their ecological impact. The social insects, mainly include ants, termites, social bees, and social wasps, often form large groups with an extraordinary high local density, and they make up more than 75% of the biomass in the Amazon rain forest (Hölldobler & Wilson, 1990). Therefore, the ecological factors of group size and worker density are important factors to explain the group living of social insects and their major ecological roles. Besides, the group size and worker density affect the emergence of increased division of labor (Ulrich et al., 2018) and the level of collective organization (Dornhaus et al., 2012), which lead to the different benefits at group‐level.

Previous major studies have focused on the influence of group size on group productivity and social complexity in social insects (Bourke, 1999; Holbrook et al., 2011), few have examined the effects of worker density on group benefits. Worker density refers to the number of workers in a unit area, which is a better ecological factor than the total number of workers or group size because it considers the number of workers in a finite environment. The inhabit nest of social insects usually contains thousands of individuals, of which physical and chemical interactions facilitate information flows and collective behaviors (e.g., foraging, colony defense, and nest moving; Cao, 2013). Changes of individual space and intranidal crowding would alter social interactions, such as allocating tasks and working load (Cao et al., 2007; O’Donnell & Bulova, 2007). Thus, changes in worker density should affect individuals’ communication and cooperation activities, and consequently, it should influence the group benefits.

Extensive empirical evidences have mainly shown that the worker density influences the disease spread and therefore affects the reproduction and survival of the group population (Liu et al., 2015; Meunier, 2015; Pie et al., 2004). Yet, there is little demonstrating that group benefits directly increased along with worker density, as we have shown here. Our experiments firstly explored the relationship between worker density, quantity of eggs laid, and total biomass accumulation from group perspective. The number of eggs in the group is a measurement of group reproduction. The total biomass accumulation, which not only contains the biomass change of living individuals, but also the biomass loss followed by death numbers, is a better indicator than mortality, to be used as a measurement of the viability of the group during a specific period of time. In many organisms, fertility and viability are negatively correlated, increasing reproduction exacts costs in terms of reduced maintenance (e.g., stress resistance and immunity) in lowered survival (Flatt, 2011; Harshman & Zera, 2007). However, our study suggests that at the group level, as the worker density increases, the original negative correlation between fertility and viability of group benefits is broken, and both increase.

Furthermore, our results consequently show that groups of the termite *R*. *chinensis* laid more eggs and accumulated more biomass in a small living area than in a large living area, even if they consume the same quantity of resource. These results suggest that workers in *R*. *chinensis* may improve the utilization of resources in higher worker density. Changes in worker density or colony size can influence individual‐ and colony‐level physiological and behavioral traits and thus affect the efficiency and productivity of the colony (Anderson & McShea, 2001; Karsai & Wenzel, 1998). Like in *Temnothorax* ants, it has been showed that in larger laboratory nests, which contain less individuals in a unit area, individuals consume proportionally less food and have less per capita brood production (Cao & Dornhaus, 2013). Moreover, the spider *Stegodyphus dumicola* may get benefits like increased feeding efficiency and lower mass loss with group size (Vanthournout et al., 2016). One potential explanation about our results is that the interaction and communication between group members become more frequent and more convenient in higher worker density, thus, the transmission of materials and the share of food become more efficient among group members. In addition, workers would be more efficient to remove pathogens in smaller areas which reduces the spread of diseases and increases the efficiency of group members’ work (Liu et al., 2019, 2020).

Although our results have demonstrated that worker density is an important factor that drives group reproduction in the termite *R*. *chinensis*, relatively few behavioral mechanisms are known on the way it influences. Usually, the queens only engage in reproduction, they do not feed directly. Their nutritional materials are mainly provided by workers, and the way to provide is by the behavior of trophallaxis. The trophallaxis is a transfer of alimentary liquids, including suspended particulates and derivatives, from one individual to another via regurgitation (stomodeal trophallaxis) or anal feeding (proctodeal trophallaxis; Huang et al., 2008; Mcmahan, 1966; Suárez & Thorne, 2000), and it is a characteristic behavior of termites and other eusocial insects for maintaining colony nutritional dynamics and pheromonal communication. Here, in the termite *R*. *chinensis*, the queens’ nutritional needs are coming mainly from workers’ stomodeal trophallaxis, so we studied stomodeal trophallaxis behavior from any workers to queens to measure the intra‐group cooperation. Our results show that workers conducted a significant higher number of stomodeal trophallaxis occurrences to queens in the worker density conditions of 0.81 worker/cm^2^ than in the worker density of 0.36 worker/cm^2^, but we did not observe any significant difference between the worker density conditions of 2.39 and 0.81 worker/cm^2^. One possible explanation is that the number of stomodeal trophallaxis occurrences from workers to the queen has reached a maximum threshold in the conditions with 0.81 worker/cm^2^. In general, our results support that the increased worker density affects the queens’ fertility in the colony by increasing intra‐group cooperation behaviors among different castes.

Termites usually live with large group size, which are made of many altruistic individuals (Abe, 1990; Howard & Thorne, 2010). How they are maintained has long been puzzled and argued in evolutionary biology (Thorne, 1997; Wilson & Wilson, 2007). Here, our findings suggest that the worker density affects the number of eggs and the total biomass accumulation could explain the ecological success of group living termites and probably of other eusocial insects.

## CONFLICT OF INTEREST

The authors declare no competing interests.

## AUTHOR CONTRIBUTION

**Zhuangdong Bai:** Conceptualization (lead); Data curation (equal); Investigation (equal); Methodology (lead); Resources (equal); Software (lead); Visualization (lead); Writing‐original draft (lead). **Yibin Liu:** Conceptualization (supporting); Data curation (equal); Investigation (equal); Methodology (supporting); Resources (equal). **David Sillam‐Dussès:** Conceptualization (supporting); Methodology (supporting); Visualization (supporting); Writing‐original draft (supporting); Writing‐review & editing (lead). **Rui‐Wu Wang:** Conceptualization (supporting); Data curation (supporting); Funding acquisition (lead); Investigation (supporting); Methodology (supporting); Project administration (lead); Resources (supporting); Software (supporting); Writing‐original draft (supporting); Writing‐review & editing (supporting).

### OPEN RESEARCH BADGES

This article has earned an Open Data Badge for making publicly available the digitally‐shareable data necessary to reproduce the reported results. The data is available at https://doi.org/10.5061/dryad.1vhhmgqr5.

## Supporting information

Tables S1‐S3

## Data Availability

Data associated with this manuscript will be available from the Dryad Digital Repository (https://doi.org/10.5061/dryad.1vhhmgqr5).
