# Supplementary material for: Experimentally measured group direct benefits according to worker density explain group living of the termite Reticulitermes chinensis
Source: Ecol Evol. 2021 Jun 16;11(13):8768–75. doi: 10.1002/ece3.7685 (PMC8258223; doi:10.1002/ece3.7685)
Supplement: Supplementary file 1 — Tables S1‐S3 [file ECE3-11-8768-s001.docx]

Supplementary information

Data S1 Data on worker density-dependent results: the group benefits with different treatments in artificial groups (in supplementary data).

Data S2 Number of stomodeal trophallaxis occurrences between any workers and the queen in artificial groups (in supplementary data).

| Set A: group members in 6-cm Petri dishes | | | | | | |
| --- | --- | --- | --- | --- | --- | --- |
| Number of individuals | 25 | 50 | 75 | 100 | 125 | 150 |
| Number of soldiers | 1 | 2 | 3 | 4 | 5 | 6 |
| Number of replacement king | 1 | 1 | 1 | 1 | 1 | 1 |
| Number of replacement queen | 1 | 1 | 1 | 1 | 1 | 1 |
| Worker density (individuals/cm^2^) | 0.78 | 1.63 | 2.48 | 3.32 | 4.17 | 5.02 |

Table S1 Groups of set A and B with the different quantity intervals in the worker density control experiments. The composition of groups at followings:

| Set B: group members in 9-cm Petri dishes | | | | | |
| --- | --- | --- | --- | --- | --- |
| Number of individuals | 50 | 100 | 150 | 200 | 250 |
| Number of soldiers | 2 | 4 | 6 | 8 | 10 |
| Number of replacement king | 1 | 1 | 1 | 1 | 1 |
| Number of replacement queen | 1 | 1 | 1 | 1 | 1 |
| Worker density (individuals/cm^2^) | 0.72 | 1.48 | 2.23 | 2.99 | 3.74 |


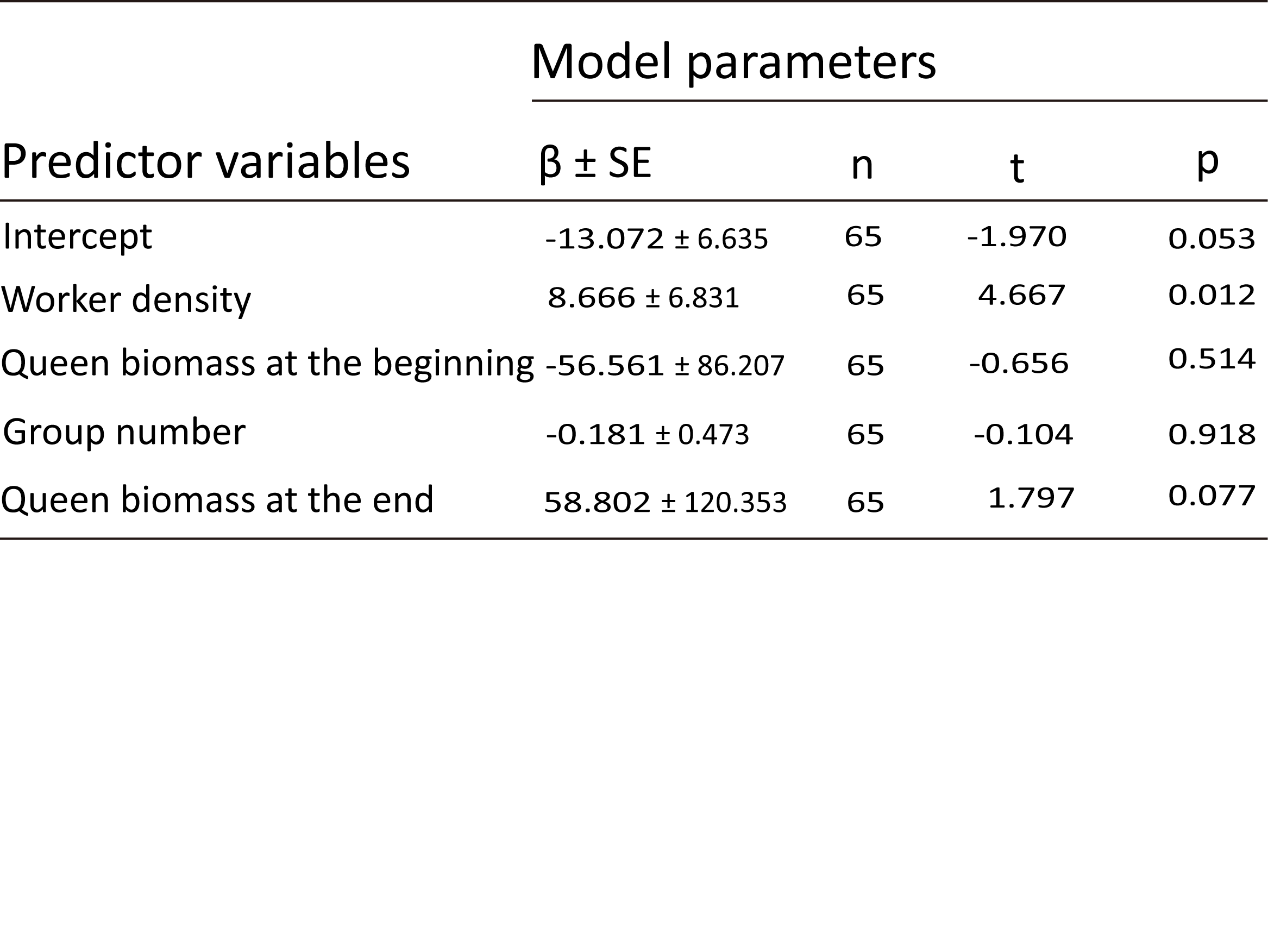


Table S2 Results of four variables that might influence the number of eggs in the group, which were performed by multivariate analysis using the linear mixed models, with colony was set as random effect.


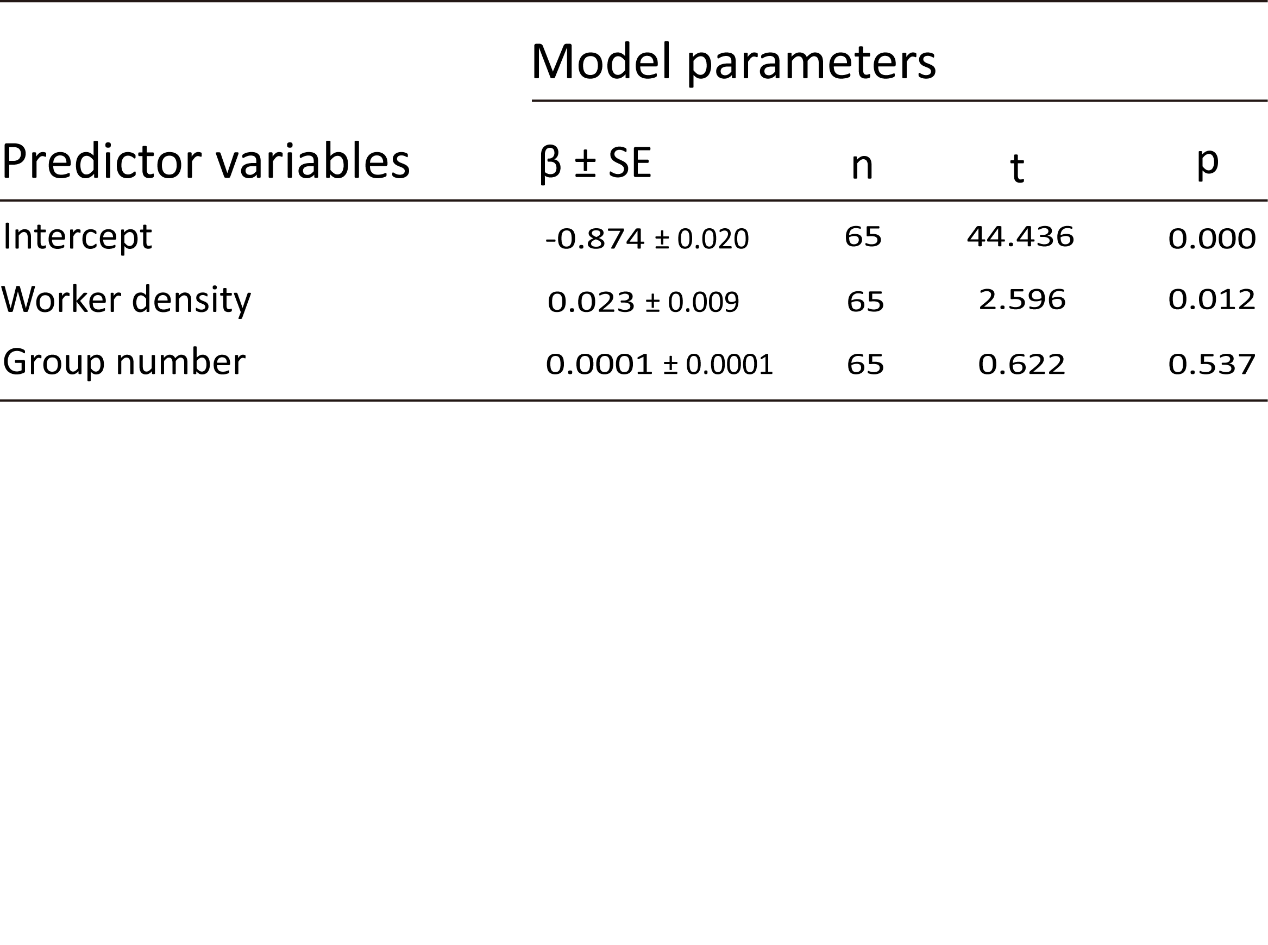


Table S3 Results of two variables with the worker density and group number that might influence the ratio of total biomass accumulation in the group, which were performed by multivariate analysis using the linear mixed models, with colony was set as random effect.
